# Increased physiological dead space in mechanically ventilated COVID-19 patients recovering from severe acute respiratory distress syndrome: a case report

**DOI:** 10.1186/s12879-020-05360-5

**Published:** 2020-08-27

**Authors:** Jingen Xia, Yingying Feng, Min Li, Xin Yu, Yi Zhang, Jun Duan, Qingyuan Zhan

**Affiliations:** 1 Department of Pulmonary and Critical Care Medicine, National Clinical Research Center for Respiratory Diseases, Institute of Respiratory Medicine, Chinese Academy of Medical Sciences, China-Japan Friendship Hospital, No 2, East Yinghua Road, Chaoyang District, Beijing, 100029 China; 2 School of Biological Science and Medical Engineering, Beihang University, Beijing, 100191 China; 3 Department of Surgical Intensive Care Unit, China-Japan Friendship Hospital, Beijing, 100029 China

**Keywords:** Coronavirus disease 2019, Acute respiratory distress syndrome, Dead space ventilation, Mechanical ventilation, Case report

## Abstract

**Background:**

An ongoing outbreak of coronavirus disease 2019 (COVID-19) is spreading globally. Recently, several articles have mentioned that the early acute respiratory distress syndrome (ARDS) caused by COVID-19 significantly differ from those of ARDS due to other causes. Actually, we newly observed that some mechanically ventilated COVID-19 patients recovering from severe ARDS (more than 14 days after invasive ventilation) often experienced evidently gradual increases in CO_2_ retention and minute ventilation. However, the underlying mechanics remain unclear.

**Case presentation:**

To explain these pathophysiological features and discuss the ventilatory strategy during the late phase of severe ARDS in COVID-19 patients, we first used a metabolic module on a General Electric R860 ventilator (Engstrom Carestation; GE Healthcare, USA) to monitor parameters related to gas metabolism, lung mechanics and physiological dead space in two COVID-19 patients. We found that remarkably decreased ventilatory efficiency (e.g., the ratio of dead space to tidal volume 70–80%, arterial to end-tidal CO_2_ difference 18–23 mmHg and ventilatory ratio 3–4) and hypermetabolism (oxygen consumption 300–400 ml/min, CO_2_ elimination 200–300 ml/min) may explain why these patients experienced more severe respiratory distress and CO_2_ retention in the late phase of ARDS caused by COVID-19.

**Conclusion:**

During the recovery period of ARDS among mechanically-ventilated COVID-19 patients, attention should be paid to the monitoring of physiological dead space and metabolism. Tidal volume (8–9 ml/kg) could be increased appropriately under the limited plateau pressure; however, barotrauma should still be kept in mind.

## Background

An ongoing outbreak of coronavirus disease 2019 (COVID-19) is spreading globally [1, 2]. As of April 29, 2020, the number of total confirmed cases has exceeded 3 million, associated to 207,973 deaths worldwide [1]. Recently, several articles [3–5] have reported that the early acute respiratory distress syndrome (ARDS) caused by coronavirus disease 2019 (COVID-19) significantly differ from those of ARDS due to other causes, such as mismatch between changes in respiratory mechanics and severity of impaired oxygenation [3], significantly decreased ventilation efficiency [4], and lower lung recruitability [5]. We also found that many mechanically ventilated COVID-19 patients recovering from severe ARDS experienced gradual increases in CO_2_ retention and minute ventilation (MV). To explain these pathophysiological features and discuss the ventilatory strategy during the late phase of severe ARDS in COVID-19 patients, we first used a metabolic module (COVX module; GE Healthcare, Helsinki, Finland) on a General Electric R860 ventilator (Engstrom Carestation; GE Healthcare, USA) to monitor parameters related to gas metabolism, end-expiratory lung volume (EELV), physiological dead space, ventilatory ratio (VR) [4], and lung mechanics in two COVID-19 patients.

## Case presentation

**Fig. 1:**
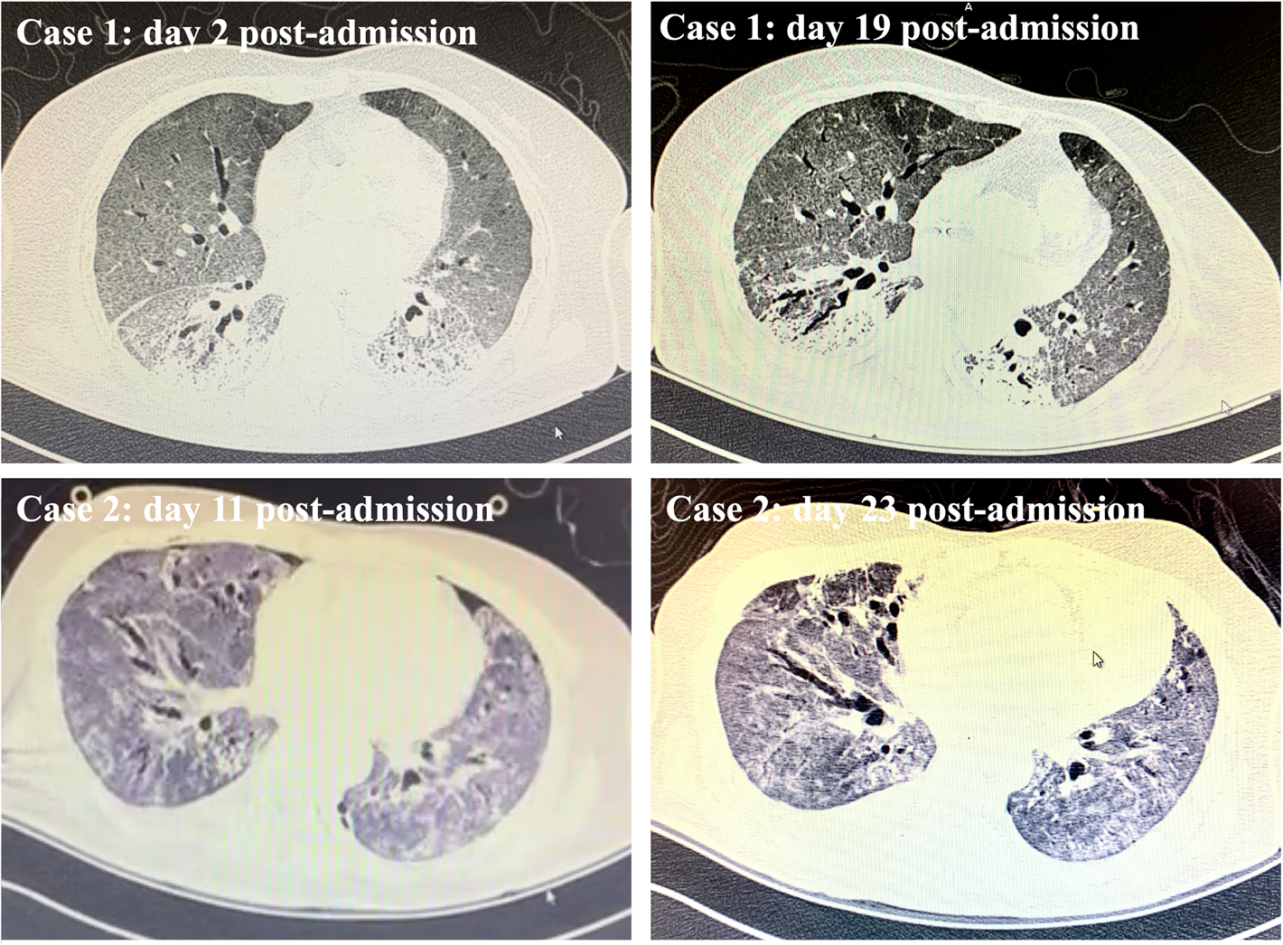
Left: transverse chest CT images from a 70-year-old female COVID-19 patient showing bilateral diffuse ground glass opacity, interstitial fibrosis, traction bronchiectasis and a small amount of lung consolidation in the dependent lungs on 2 and 19 days after invasive ventilation. Right: transverse chest CT images from a 42-year-old male COVID-19 patient showing bilateral ground glass opacity, interstitial fibrosis, left pleural effusion and traction bronchiectasis on 36 days and 48 days after invasive ventilation

Case 1: A 70-year-old woman (BMI, 21.5 kg/m^2^) with acute respiratory failure caused by COVID-19 was transferred to Tongji Hospital (Wuhan, China) on February 26, 2020. On admission, she had severe dyspnea (respiratory rate [RR], 40 bpm) and acute hypoxemic respiratory failure (oxygen saturation 45% with oxygen flow 12 L/min) and underwent endotracheal intubation immediately. A tracheotomy was performed 6 days post-admission. She was supported in pressure control mode: inspiratory pressure 15–18 cmH_2_O and positive end-expiratory pressure (PEEP) 6–8 cmH_2_O. About 14 days post-admission, she experienced a gradual increase in RR (from 25 to 30–40 bpm), tidal volume (VT, from 410 to 480 ml [7.6–9.1 ml/kg PBW]), and MV (from 10 to 12–15 L/min), accompanied by an increased PaCO_2_ (from 39 to 47–60 mmHg) and decreased oxygenation (arterial partial pressure of oxygen [PaO2]/fraction of inspired oxygen [FiO2], from 127 to 74–100 mmHg; FiO_2_, from 0.5 to 0.7). On day 15, the ventilator was changed to an R860 ventilator with COVX module. The following data were recorded: oxygen consumption (VO_2_) 280 ml/min, CO_2_ elimination (VCO_2_) 175 ml/min; ratio of dead space to VT (VD/VT) 78%, end-tidal carbon dioxide (ETCO_2_) 26.1 mmHg, arterial to end-tidal CO_2_ difference (P_(a-ET)_CO_2_) 22.3 mmHg, VR 3.57; EELV 1672 ml; airway resistance (R) 10.8 cmH_2_O/L/s, and respiratory system compliance (Cresp) 12.5 ml/cmH_2_O. Chest computed tomography (CT) revealed bilateral diffuse ground glass opacity, interstitial fibrosis, traction bronchiectasis, and a small amount of consolidation in the dependent aspects of the lungs on 2 day and 19 day after admission (Fig. 1). Forty days after admission, her lung function still did not recover from sustained hypercapnia in the treatment of Lopinavir/ritonavir, infusion of convalescent plasma, low molecular weight heparin and prone position ventilation, and was transferred to another hospital.

Case 2: A 42-year-old man (BMI, 22.9 kg/m^2^) diagnosed with COVID-19 was transferred to Tongji Hospital (Wuhan, China) on March 3, 2020. Tracheal intubation and venous-venous extracorporeal membrane oxygenation (ECMO) were performed 25 and 24 days, respectively, before admission due to severe refractory ARDS. ECMO catheters were removed after 14 days of treatment due to a severe blood stream infection. However, ECMO was performed again due to bilateral pneumothorax 8 days before admission. Post-admission, he was treated with antiviral therapy, antibiotic therapy, tracheotomy (1 day post-admission), prone ventilation, and other treatments using our standard protocol. Oxygenation improved gradually, and the ECMO catheter was removed again 19 days post-admission. Over the next week, he showed a gradual increase in MV and hypercapnia. On day 26 post-admission, he was ventilated mechanically in pressure support mode: pressure support 18 cmH_2_O, PEEP 3 cmH_2_O, FiO_2_ 0.3, VT 651 ml (9.2 ml/kg), RR 31 bpm, and MV 17.9 L/min. Arterial blood gas revealed the following: pH 7.463, PaCO_2_ 51 mmHg, PaO_2_ 80.4 mmHg, HCO_3_ 34.9 mmol/L, and PaO2/FiO2 268 mmHg. The following data were recorded using the COVX module: VO_2_ 401 ml/min, VCO_2_ 292 ml/min; VD/VT 76%, ETCO_2_ 33.2 mmHg, P_(a-ET)_CO_2_ 17.8 mmHg, VR 3.4; EELV 1000 ml; R 7.7 cmH_2_O/L/s and Cresp 19.6 ml/cmH_2_O. Chest CT showed bilateral ground glass opacity, interstitial fibrosis, and traction bronchiectasis 11 days and 23 days post-admission (Fig. 1). He was disconnected from invasive ventilator and transferred to another hospital for further pulmonary rehabilitation on 38 days post-admission.

## Discussion and conclusion

We found that the lung tissue of COVID-19 patients recovering from severe ARDS not only reflects the typical characteristics of late-phase ARDS (reduced lung compliance, pulmonary fibrosis, and decreased EELV) but is also associated with a significantly increased dead space (VD/VT 70–80%, VR 3–4), markedly higher than that in patients with severe ARDS due to other reasons [6]. Besides, some COVID-19 patients show an obvious hypermetabolic status even in the recovery period. Therefore, significantly decreased ventilation efficiency and hypermetabolism may explain why these patients experienced more severe respiratory distress and CO_2_ retention in the late phase of ARDS.

A remarkably increased physiological dead space may be a prominent pathophysiological feature in mechanically ventilated COVID-19 patients recovering from severe ARDS; however, the underlying mechanism remains unclear. It may be related to significant regional ventilation/perfusion heterogeneity [7] due to loss of lung perfusion regulation and hypoxic vasoconstriction [3], pulmonary microthrombosis [8], and increased anatomical dead space from obvious traction bronchiectasis observed on chest CT.

To reduce the risk of ventilator-induced lung injury with high tidal volume (8–9 ml/kg), we tried to reduce the VT (6–8 ml/kg) and increase the RR to maintain the MV. However, severe hypercapnia (PaCO_2_ 98 mmHg, pH 7.10 in case 2) was observed, and the use of sedatives and anesthetic agents had to be increased. These serious consequences are worthy of attention. In these two patients, we set a higher VT (8–9 ml/kg), lower peak airway pressure (< 25 cmH_2_O), and low PEEP levels (3–6 cmH_2_O) due to low lung recruitability; barotrauma did not occur. Of course, our experience from few patients cannot be extrapolated to all COVID-19 patients, however, this report provides a possible more suitable lung protective ventilation for COVID-19 patients recovering from acute respiratory failure with refractory hypercapnia, which deserved to be further investigated in clinical research.

In conclusion, during the recovery period of ARDS in mechanically ventilated COVID-19 patients, attention should be paid to the monitoring of physiological dead space and VR [4]. Tidal volume (8–9 ml/kg) could be increased appropriately under the limited plateau pressure; however, barotrauma should still be considered. In theory, extracorporeal CO_2_ removal is a better choice for these patients [9] and will be more beneficial to reduce lung injury while awaiting lung tissue repair; however, further clinical investigations are warranted.

## Data Availability

Not applicable.

## Abbreviations

ARDS: Acute respiratory distress syndrome
Cresp: Respiratory system compliance
COVID-19: Coronavirus disease 2019
CT: Computed tomography
ECMO: Extracorporeal membrane oxygenation
EELV: End-expiratory lung volume
FiO_2_: Fraction of inspired oxygen
MV: Minute ventilation
PaCO_2_: Arterial partial pressure of carbon dioxide
P_(a-ET)_CO_2_: Arterial to end-tidal CO_2_ difference
PaO_2_: Arterial partial pressure of oxygen
PEEP: Positive end-expiratory pressure
R: Airway resistance
RR: Respiratory rate
VD/VT: Ratio of dead space to VT
VR: Ventilatory ratio
VT: Tidal volume
VO_2_: Oxygen consumption
VCO_2_: CO_2_ elimination
